# GLP-1 selectively enhances tonic GABA_A_ receptor-mediated currents in mouse dentate gyrus granule cells of the ventral hippocampus

**DOI:** 10.3389/fncel.2025.1638550

**Published:** 2025-10-01

**Authors:** Olga Netsyk, Sergiy V. Korol, Bryndis Birnir, Zhe Jin

**Affiliations:** Department of Medical Cell Biology, Uppsala University, Uppsala, Sweden

**Keywords:** GABA, inhibition, GABA_A_ receptor, glucagon-like peptide-1, hormone

## Abstract

Glucagon-like peptide-1 (GLP-1) is a metabolic hormone secreted by L-cells in the gut and it stimulates insulin secretion in the pancreatic islets by activating GLP-1 receptors (GLP-1Rs). In the brain, the GLP-1Rs are expressed in many regions including the hippocampus. We examined whether GLP-1 modulation of GABA-activated currents in the mouse hippocampus varied along the hippocampal dorsal-ventral axis. We recorded spontaneous inhibitory postsynaptic (sIPSCs) and tonic extrasynaptic currents in dorsal and ventral hippocampal dentate gyrus (DG) granule cells in brain slices from 2-month-old mice. GLP-1 (100 pM) did not modulate the GABA-activated fast or slow phasic postsynaptic currents in either the dorsal or the ventral hippocampal slices. In contrast, the tonic extrasynaptic current was potentiated by GLP-1 but, only consistently in the DG granule cells of the ventral hippocampus. Thus, GLP-1 modulation of the DG neurons depends on the dorso-ventral longitudinal hippocampal axis and further, with the subcellular location (synaptic vs. extrasynaptic) of the GABA_A_ receptors (GABA_A_R) in the DG granule cells. The results are consistent with GLP-1 enhancing the tonic inhibitory extrasynaptic current by a postsynaptic mechanism.

## Introduction

The incretins are secreted in response to ingestion of food, and it is well established that they regulate insulin secretion in a glucose-dependent manner (Astrup, 2024; Muller et al., 2019). GLP-1 is one of the incretins hormones that are secreted by cells in the gut. Moreover, GLP-1 and GLP-1Rs are also found in the brain where they have been ascribed various functions including neuroprotection, modulating food intake and enhancing memory and learning in the hippocampus (Astrup, 2024; Gupta et al., 2023; Holscher, 2022; Kanoski and Grill, 2017). Nucleus of the solitary tract (NTS) is the primary neuronal source of the endogenous GLP-1 in the brain (Larsen et al., 1997) where the preproglucagon is processed to GLP-1 (Ugleholdt et al., 2004). However, axons from NTS do not project to the hippocampus. How GLP-1 reaches the hippocampus is still under investigation, but it has been suggested that GLP-1 may reach the hippocampus by simple diffusion or through volume transmission from the ventricular system (Gupta et al., 2023; Buller and Blouet, 2024; Hsu et al., 2015; Kanoski et al., 2016; Kastin et al., 2002). Both the dorsal and the ventral DG regions of the mouse hippocampus express GLP-1R (Holst, 2024). In recent years glucagon-like peptide-1 receptor agonists (GLP-1RAs) have been shown in clinical studies to be neuroprotective, to have metabolic benefits and have emerged as effective treatments for both type 2 diabetes and obesity (Astrup, 2024; Dash, 2024), while effects related to decreasing the rate of cognitive decline are not as clear and are still being explored (Dash, 2024; Liang et al., 2024).

The hippocampal longitudinal axis ranges from dorsal (septal) to ventral (temporal) in rodents and corresponds to posterior-to-anterior hippocampus in humans (Papatheodoropoulos, 2018; Strange et al., 2014). Information from sensory cortices is received by the dorsal hippocampus whereas the ventral hippocampus has more connectivity with the amygdala, prefrontal cortex and hypothalamus (Strange et al., 2014; Swanson and Cowan, 1977). Hippocampal activity is modulated, at least in part, by hormones, as the expression of many hormone receptors has been detected (Lathe, 2001). The role of the hippocampus in formation of spatial memories, navigation and emotional responses is well established (Strange et al., 2014) but what is less well known is that the hippocampus participates in regulating physiological homeostasis in a topographical manner (Risold and Swanson, 1996). The ventral hippocampal neurons, for instance, via a synapse in the septum, inhibit hypothalamic neurons (Risold and Swanson, 1996; Decarie-Spain et al., 2022). It is, therefore, not surprising that metabolic hormones like insulin and GLP-1 have been shown to modulate synaptic transmission in hippocampal neurons (Ferrario and Reagan, 2018; Hammoud et al., 2021; Korol et al., 2015).

**Figure 1:**
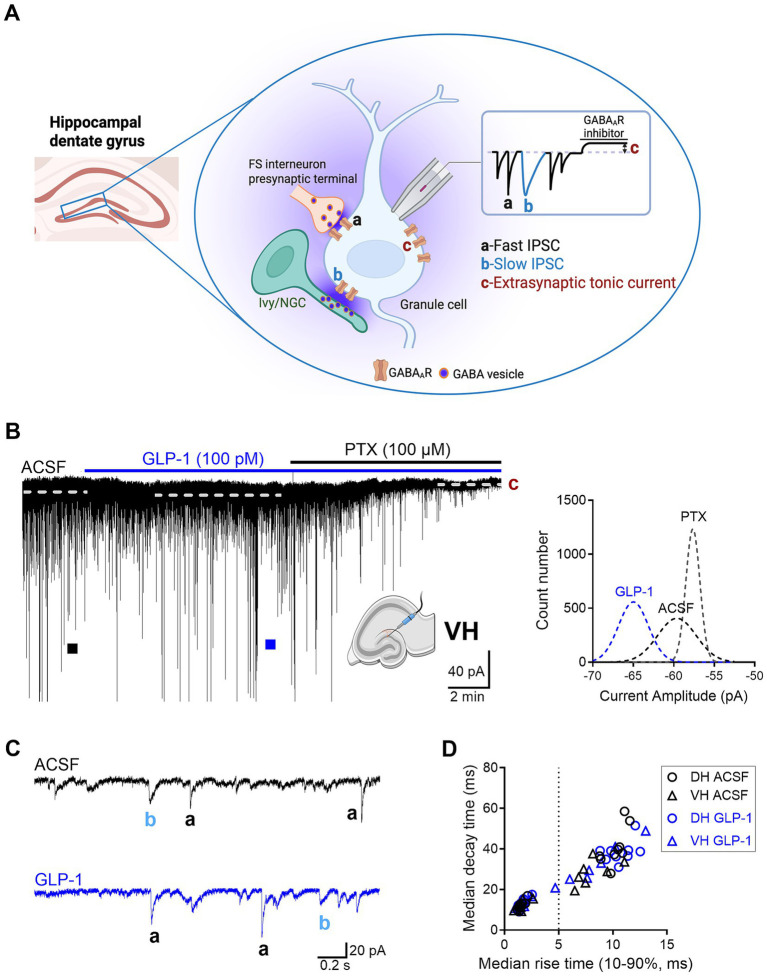
The effect of GLP-1 on GABA_A_R-mediated currents in dentate gyrus granule cells of the mouse hippocampus. **(A)** A schematic illustration of three forms of GABA_A_R-mediated currents recorded in dentate gyrus (DG) granule cells: a, fast inhibitory postsynaptic current (IPSC); b, slow IPSC; c, extrasynaptic tonic current. FS interneuron, fast-spiking interneuron; Ivy/NGC, Ivy/neurogliaform cell. Created in BioRender. Jin (2025), https://BioRender.com/g5wydyv. **(B)** A representative current trace recorded from a DG granule cell in the ventral hippocampus (VH) before and after GLP-1 (100 pM) application. The difference between the dashed lines represents the extrasynaptic tonic current amplitude (c), estimated from Gaussian fits to all-points histograms derived from sIPSC-free baseline segments (right panel)**. (C)** Fast IPSC (a) and slow IPSC (b) from segments marked with filled squares are shown on an expanded scale below. ACSF, artificial cerebrospinal fluid; PTX, picrotoxin. **(D)** A scatter plot illustrating the median 63% decay time plotted against the median 10–90% rise time of sIPSCs measured in individual DG granule cells (DH, *n* = 9 from 6 mice; VH, *n* = 7 from 5 mice). sIPSCs were classified as fast or slow based on a 5-ms rise-time cutoff.

*γ*-Aminobutyric acid (GABA) is the main inhibitory neurotransmitter in the central nervous system (Sieghart and Savic, 2018). It activates GABA_A_ and GABA_B_ receptors that are ion channels and G-protein coupled receptors, respectively. When GABA is released from presynaptic terminals it activates synaptic GABA_A_ receptors (GABA_A_Rs) on postsynaptic neurons generating phasic spontaneous inhibitory postsynaptic currents (sIPSCs) (Otis et al., 1991). These phasic currents comprise both fast and slow sIPSCs (Figure 1A), which are distinguished not only by their kinetics but also by their distinct presynaptic GABAergic neurons that evoke them, and play different roles in neuronal circuits (Armstrong et al., 2012; Capogna and Pearce, 2011). GABA_A_Rs located outside of synapses are termed extrasynaptic receptors and are activated by ambient GABA concentrations and mediate extrasynaptic tonic current (Figure 1A) (Semyanov et al., 2003). GABA_A_Rs containing α4β2/3δ mainly mediate tonic inhibition in mouse DG granule cells (Chandra et al., 2006; Stell et al., 2003). We have previously shown in rat dorsal hippocampal CA3 neurons that GLP-1 and its mimetics enhanced both synaptic and extrasynaptic GABA-activated currents (Korol et al., 2015; Babateen et al., 2017; Korol et al., 2015). Here, we examine whether GLP-1 differentially modulates GABAergic inhibition in mouse dorsal and ventral hippocampal DG granule cells. In line with common simplification of the endogenous diversification of the hippocampus, we divided the structure along the dorsoventral axis into dorsal, intermediate and ventral domains (Strange et al., 2014; Paxinos and Watson, 1986). Our previous work has shown that GABAergic inhibition in the mouse hippocampus varies and is dependent on the dorsoventral axis and cell type (DG granule cells and CA3 pyramidal neurons) (Netsyk et al., 2020). We then studied the effects of GLP-1 (100 pM) on GABAergic signaling in dorsal and ventral dentate gyrus (DG) granule cells. The results show that GLP-1 modulates the GABAergic currents mediated via extrasynaptic GABA_A_ receptors in DG granule cells only in the ventral part of the mouse hippocampus.

## Materials and methods

### Animals

All experiments were conducted in accordance with the local ethical guidelines and protocols approved by Uppsala Animal Ethical Committee, Swedish law and regulations based on the Directive 2010/63/EU and C129/14. C57BL/6 J male mice (Taconic M&B, Denmark), aged 8–10 weeks, were used in all experiments. Recordings were made from DG granule cells in hippocampal dorsal and ventral brain slices.

### Hippocampal slice preparation

Mice were euthanized by cervical dislocation followed by decapitation. Brain slices were prepared as previously described (Netsyk et al., 2020; Jin et al., 2011; Ting et al., 2014). Briefly, the brain was removed and placed into ice-cold N-methyl D-glucamine (NMDG)-based solution containing (mM): 93 NMDG, 2.5 KCl, 1.2 NaH_2_PO_4_, 30 NaHCO_3_, 20 4-(2-hydroxyethyl)piperazine-1-ethanesulfonic acid (HEPES), 25 D-glucose, 10 MgSO_4_, 0.5 CaCl_2_, 5 Na ascorbate, 2 thiourea, 3 Na pyruvate, pH 7.3–7.4 (adjusted with HCl), saturated with 95% O_2_ and 5% CO_2_, osmolarity 300–305 mOsm (adjusted with sucrose). Hippocampal slices (350 μm thick) were cut using a microtome (Leica VT1200 S, Leica Microsystems AB, Germany). Dorsal and ventral DG were defined in coronal and horizontal slices, respectively, according to Paxinos and Watson (1986). The slices were incubated in NMDG-based solution at 32 °C for 12–15 min, then transferred to a HEPES-based holding solution (in mM): 92 NaCl, 2.5 KCl, 1.2 NaH_2_PO_4_, 30 NaHCO_3_, 20 HEPES, 25 D-glucose, 2 MgSO_4_, 2 CaCl_2_, 5 Na ascorbate, 2 thiourea, 3 Na pyruvate, pH 7.3–7.4 (adjusted with NaOH), saturated with 95% O_2_ and 5% CO_2_; osmolarity 300–305 mOsm (adjusted with sucrose). The slices were kept at room temperature (20–22 °C) for at least 1 h before use.

### Electrophysiology

Whole-cell patch-clamp recordings were performed on DG granule cells from the dorsal and ventral regions of the hippocampus (Netsyk et al., 2020). All experiments were conducted at room temperature. The slice was transferred to the recording chamber and perfused (1.5–2 mL/min) with artificial cerebrospinal fluid (ACSF) containing (mM): 119 NaCl, 2.5 KCl, 1.3 MgSO_4_, 1 NaH_2_PO_4_, 26.2 NaHCO_3_, 2.5 CaCl_2_, 11 D-glucose and 3 kynurenic acid, pH 7.3–7.4, equilibrated with 95% O_2_ and 5% CO_2_, osmolarity 300–303 mOsm (adjusted with sucrose). Borosilicate glass patch pipettes (4–5 MΩ in resistance) were filled with an intracellular solution containing (mM): 140 CsCl, 8 NaCl, 2 EGTA, 0.2 MgCl_2_, 10 HEPES, 2 MgATP, 0.3 Na_3_GTP, 5 QX314Br, pH 7.2 (adjusted with CsOH), osmolarity 285–290 mOsm. The order of DH and VH recordings was randomized. The experimenter was not blinded to treatment due to the pre−/post-application design. Data collection began approximately 7–10 min after achieving whole-cell configuration. sIPSCs were recorded for ≥ 5 min after baseline stabilization and ≥ 8 min during GLP-1 application. Picrotoxin (100 μM) was applied to block GABA_A_R and reveal extrasynaptic tonic currents. Kynurenic acid (3 mM) was added to block glutamatergic synaptic transmission. Voltage-clamp current recordings were made at −60 mV holding potential, filtered at 2 kHz using a Multipatch 700B amplifier and Axon Digidata board 1550A, controlled by pCLAMP 10.5 software (Axon Instruments, Molecular Devices, CA, USA).

### Drugs

GLP-1 (7-36) amide, human was purchased from Anaspec (AS-22462, Anaspec Europe, Belgium); other chemicals were from Sigma-Aldrich (Steinheim, Germany).

GLP-1 lyophilized powder was reconstituted in distilled water as a stock solution, aliquoted, and stored at −20 °C. Each aliquot was then thawed once and diluted in ACSF immediately before use.

### Data analysis

The currents were analyzed as described previously (Netsyk et al., 2020; Netsyk et al., 2025). The membrane capacitance of DG granule cells in the DH was significantly lower than in the VH (DH, 54.1 ± 3.6 pF, n = 9; VH, 68.9 ± 4.9 pF, *n* = 7; unpaired Student’s *t* test, *p* = 0.026), consistent with our previous study (Netsyk et al., 2020). The average access resistance (R_a_) did not differ in DG granule cells between DH and VH (DH, 42.33 ± 3 0.84 MΩ, n = 9; VH, 38.94 ± 6.45 MΩ, *n* = 7; unpaired Student’s t test, *p* = 0.64). R_a_ was monitored throughout each recording, and recordings with >25% change in R_a_ were excluded from analysis. Briefly, sIPSCs were analyzed using MiniAnalysis software 6.0 (Synaptosoft, Decatur, GA, USA). sIPSC events were detected if larger than a threshold value set as 5xRMS (root-mean-square of the baseline noise) and visually inspected. RMS baseline noise was similar in DG granule cell recordings from both DH and VH (DH, 1.85 ± 0.14 pA, n = 9; VH, 1.99 ± 0.076 pA, *n* = 7, unpaired Student’s *t* test, *p* = 0.41). A 3–5 min segment was used for analysis. sIPSC parameters (frequency, median amplitude, 10–90% median rise time, 63% median decay time and median charge transfer) were automatically analyzed by the MiniAnalysis software. sIPSC with 10–90% rise times ≤ 5 ms were classified as fast; > 5 ms as slow (Figure 1D and Supplementary Figures 1A,B) (Netsyk et al., 2025). Tonic currents were analyzed using pCLAMP 10.5 software (Axon Instruments, Molecular Devices, San Jose, CA, USA). To determine baseline current amplitude, Gaussian fits were performed on all-points histograms derived from baseline current segments that were free of sIPSC (Figures 1B, 3D). The extrasynaptic tonic current amplitude was quantified as the shift of the baseline current after application of picrotoxin (Jin et al., 2011).

### Statistics

Data were analyzed using GraphPad Prism 10 (GraphPad Software La Jolla, CA, USA). Normality was assessed with the Shapiro–Wilk test. Paired comparisons used Student’s *t-*test (normal data) or Wilcoxon signed-rank test (non-normal data). *p*-value <0.05 was considered statistically significant.

## Results

GABA activates GABA_A_Rs to mediate various forms of inhibitory currents with specialized functional roles, including phasic currents (fast and slow sIPSCs), and extrasynaptic tonic currents. In the hippocampus, fast and slow sIPSCs are mainly evoked by GABA release from presynaptic fast-spiking interneurons and neurogliaform/Ivy cells (via volume transmission), respectively (Figure 1A) (Armstrong et al., 2012; Capogna and Pearce, 2011; Netsyk et al., 2025). Slow sIPSCs can also result from distal inputs targeting granule-cell dendrites (e.g., somatostatin-expressing interneurons, such as hilar perforant path-associated cells), where electrotonic filtering and spatial attenuation prolong rise and decay time. Outside the synapses, ambient GABA can activate high affinity extrasynaptic GABA_A_Rs, which generates persistent tonic currents (Figure 1A) (Bai et al., 2001).

**Figure 2:**
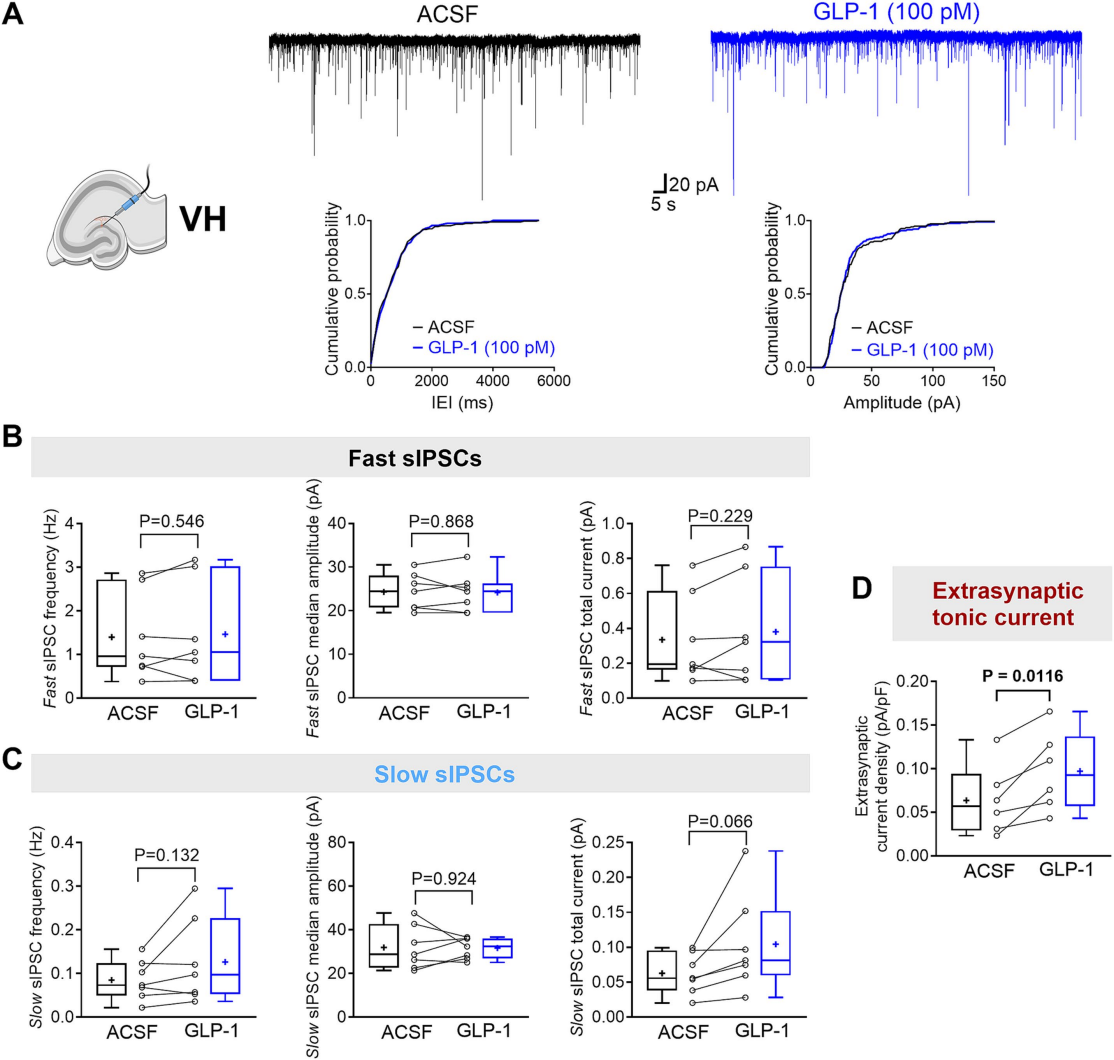
GLP-1 selectively potentiates GABA_A_R-mediated extrasynaptic tonic currents in dentate gyrus granule cells of the mouse ventral hippocampus. **(A)** The segments of representative current traces recorded from a DG granule cell in the ventral hippocampus (VH) before and after GLP-1 (100 pM) application. Cumulative probability plots for the inter-event interval (IEI) and median amplitude of fast IPSCs are shown below. ACSF, artificial cerebrospinal fluid. **(B,C)** Summary statistics for frequency, median amplitude, and total current of fast IPSC **(B)** and slow IPSC **(C)** (*n* = 7 from 5 mice). **(D)** The GABA_A_R-mediated extrasynaptic tonic current density was significantly increased after GLP-1 application (*n* = 6 from 5 mice). Data are presented as individual values with paired lines (before and after GLP-1 application), and box and whisker plots (whiskers defined by Tukey’s method). Mean values are denoted by “+.” All datasets passed the Shapiro–Wilk normality test. Statistical analysis used paired Student’s *t*-test, with *p* < 0.05 considered statistically significant.

**Figure 3:**
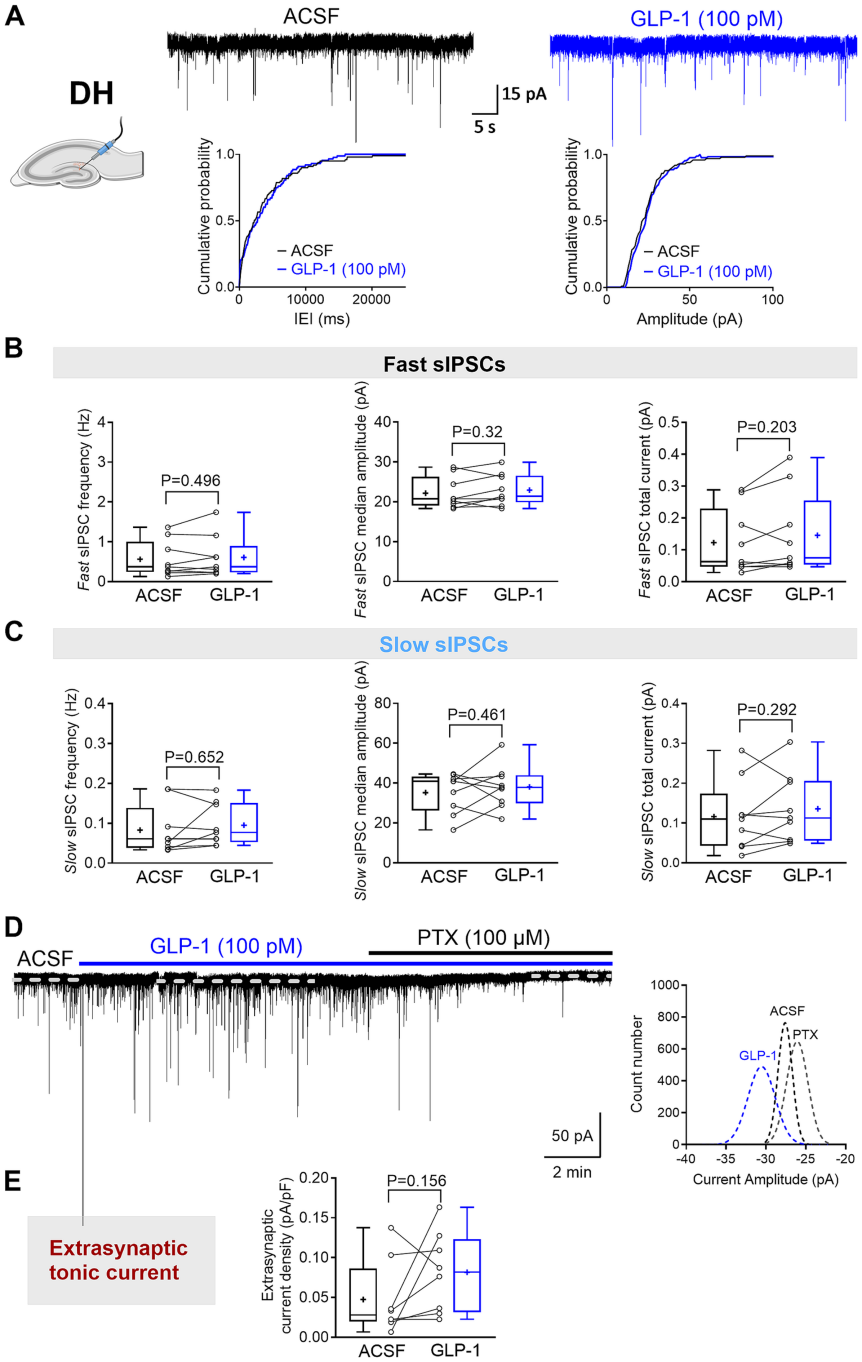
GLP-1 does not affect GABA_A_R-mediated currents in dentate gyrus granule cells of the mouse dorsal hippocampus. **(A)** The segments of representative current traces recorded from a DG granule cell in the dorsal hippocampus (DH) before and after GLP-1 (100 pM) application. Cumulative probability plots for the inter-event interval (IEI) and median amplitude of fast IPSCs are shown below. ACSF, artificial cerebrospinal fluid. **(B,C)** Summary statistics for frequency, median amplitude, and total current of fast IPSC **(B)** and slow IPSC **(C)** (*n* = 9 from 6 mice). **(D)** A representative current trace recorded from a DG granule cell in the DH before and after GLP-1 (100 pM) application. The difference between the dashed lines represents the extrasynaptic tonic current amplitude, estimated from Gaussian fits to all-points histograms derived from sIPSC-free baseline segments (right panel). **(E)** The GABA_A_R-mediated extrasynaptic tonic current density was not changed during GLP-1 application (*n* = 8 from 5 mice). Data are presented as individual values with paired lines (before and after GLP-1 application), and box and whisker plots (whiskers defined by Tukey’s method). Mean values are denoted by “+.” Only the median amplitude and total current of slow sIPSCs passed the Shapiro–Wilk normality test; all other datasets failed. For statistical analysis, a paired Student’s t-test was applied for normally distributed data, while a Wilcoxon matched-pairs sign rank test was used for non-normally distributed data. *p* < 0.05 was considered statistically significant.

Here, we investigated the effects of GLP-1 on the three types of GABA_A_R-mediated currents in mouse DG granule cells from the ventral and dorsal hippocampus. We used a low, physiologically relevant concentration of GLP-1 (100 pM), which we had previously shown to effectively modulate GABA signaling (Korol et al., 2015). Figures 1B,C illustrate typical GABA-activated currents and the effect of GLP-1 on DG granule cells from ventral mouse hippocampus. The characteristic sIPSCs were abolished by picrotoxin (100 μM), a GABA_A_R open-channel blocker, and the holding current shifted, revealing the extrasynaptic, tonic GABA-activated current present in the DG granule cells. In ventral hippocampal DG granule cells, analysis of phasic currents (fast and slow sIPSCs) revealed no changes in the frequency, median amplitude, 10–90% rise time, 63% decay time, charge transfer or total current following GLP-1 application (Figures 2A–C; Table 1). However, GLP-1 consistently enhanced the extrasynaptic tonic current (Figures 1B, 2D) in these cells (Paired Student’s *t* test, *n* = 6, *t* = 3.885, df = 5, 95% CI 0.01134 to 0.05569, *p* = 0.0116). In contrast, neither phasic currents (fast and slow sIPSCs) (Figures 3A–C; Table 1) nor tonic currents (Figures 3D,E) were affected by GLP-1 in dorsal hippocampal DG granule cells. These findings demonstrate that GLP-1 can enhance GABA-activated currents in the hippocampus, but this effect is dependent on the subcellular location and hippocampal axis location.

**Table 1:** GLP-1 effect on GABA-mediated fast and slow IPSC parameters in the dorsal and ventral hippocampal DG granule cells.

sIPSC	DH (n = 9)			VH (n = 7)		
	ACSF	+GLP-1	*p* value	ACSF	+GLP-1	*p* value
Fast sIPSC						
Rise time 10–90% (ms)	1.56 ± 0.1	1.63 ± 0.14	0.374	1.57 ± 0.2	1.62 ± 0.19	0.398
Decay time 63% (ms)	12.28 ± 0.7	12.78 ± 0.84	0.317	12.25 ± 0.94	12.81 ± 0.82	0.219
Charge transfer (fC)	215.8 ± 11.38	234.1 ± 15.39	0.088	237 ± 11.71	257.6 ± 14.45	0.095
Slow sIPSC						
Rise time 10–90% (ms)	10.25 ± 0.32	10.7 ± 0.42	0.331	8.12 ± 0.62	8.33 ± 1.04	0.809
Decay time 63% (ms)	40.82 ± 3.17	38.46 ± 1.86	0.359	28.44 ± 2.33	32.01 ± 3.75	0.133
Charge transfer (fC)	1395 ± 182.5	1418 ± 144	0.87	804.5 ± 101	911.5 ± 99.42	0.388

Data are presented as mean ± SEM. All datasets, except for decay time of slow sIPSCs in the DH, passed the Shapiro–Wilk normality test. Data collected before and during GLP-1 application (100 pM) application were compared using a paired Student’s *t*-test for normally distributed data and a Wilcoxon matched-pairs sign rank test for non-normally distributed data. *p*-value < 0.05 was considered statistically significant.

## Discussion

The hippocampus is a well-known brain structure required for learning and memory, with a particularly critical role in spatial navigation (Strange et al., 2014; Decarie-Spain et al., 2022). Importantly, the hippocampus is increasingly recognized to participate in modulation of metabolic regulation and homeostasis (Gupta et al., 2023; Lathe, 2001; Ferrario and Reagan, 2018; Hammoud et al., 2021; Netsyk et al., 2025). In DG granule cells from 2-month-old mouse hippocampus, GLP-1 only consistently enhanced the extrasynaptic GABA-activated currents in the ventral hippocampus and did not modulate the GABAergic phasic currents recorded in these cells, neither in the dorsal nor in the ventral hippocampus. Our results demonstrate selected effects of GLP-1 on mouse hippocampal GABA-activated signal transmission.

The hippocampus is a lamellar structure that is organized along the longitudinal, dorsal-ventral axis into functional domains (Papatheodoropoulos, 2018; Strange et al., 2014; Papatheodoropoulos, 2015). A variety of hormone receptors are expressed in the hippocampus (Lathe, 2001) but, receptors associated with feeding are in higher density in the ventral as compared to the dorsal hippocampus (Kanoski and Grill, 2017), including the GLP-1 receptors. The precise distribution pattern of the GLP-1 receptors varies somewhat between different species (Gupta et al., 2023; Cork et al., 2015; Graham et al., 2020; Jensen et al., 2018; Merchenthaler et al., 1999). GLP-1 releasing neurons from the nucleus of the solitary tract (NTS) do not directly innervate the hippocampus, raising the question of GLP-1’s origins in the hippocampus. But although, the hippocampus lacks GLP-1-containing axon terminals, GLP-1 has been detected in the hippocampus both in humans (Gupta et al., 2023) and in rodents (Hsu et al., 2015; Kastin et al., 2002). The GLP-1 presumably enters the hippocampal parenchyma by volume transmission from the cerebrospinal fluid or from the circulation (Gupta et al., 2023; Buller and Blouet, 2024; Hsu et al., 2015; Kastin et al., 2002). GLP-1 has been shown to enhance release of the neurotransmitters GABA or glutamate by presynaptic mechanism, but also, potentiate the GABA-activated currents in dorsal rat hippocampal neurons by a postsynaptic mechanism (Korol et al., 2015; Korol et al., 2015; Mietlicki-Baase et al., 2014; Rebosio et al., 2018; Shao et al., 2026; Wang et al., 2023). Although the GLP-1 receptor is not detected in interneurons of mouse DG (Jensen et al., 2018), it is enriched in glutamatergic mossy cells of the ventral DG, which innervate interneurons (Steiner et al., 2022). Activation of the GLP-1 receptor increases the action potential firing frequency of mossy cells, potentially leading to an indirect enhancement of GABA release from interneurons (Steiner et al., 2022). However, GLP-1 does not alter the frequency and amplitude of phasic inhibitory currents (fast and slow sIPSCs), which reflect presynaptic GABA release. This suggests that GLP-1 is unlikely to change the ambient GABA levels through spillover. Therefore, in the current study, only postsynaptic mechanism and only in the ventral DG granule cells were activated by GLP-1. This is in accordance with a study on mouse brains where the GLP-1 receptor was expressed in mature granule neurons (Graham et al., 2020). Enhanced tonic inhibition by GLP-1 in the ventral hippocampus decreases the excitability of the DG granule cells at this location.

Metabolic hormones have emerged as significant biological regulators of hippocampal functions. Hippocampal neuronal outputs map onto the hypothalamus in a topographical manner via neurons in the septum and commonly result in inhibition of hypothalamic activity (Risold and Swanson, 1996; Decarie-Spain et al., 2022; Arszovszki et al., 2014). Recent studies have identified the importance of the ventral hippocampus in regulating feeding behavior, food intake and food-directed memory (Hsu et al., 2015; Decarie-Spain et al., 2022; Hsu et al., 2018). The current results add to the mounting evidence of the functional variation between the dorsal and the ventral hippocampus. The differential effects of GLP-1 in the dorsal and ventral DG granule neurons indicates a distinct role of GLP-1 in these hippocampal regions.

This study has several limitations. First, the use of specific GLP-1R antagonists or conditional, region-specific GLP-1R knockout mouse models is needed to confirm that the observed effects on GABAergic transmission are mediated by GLP-1Rs rather than off-target actions. Second, sample sizes were relatively small and should be increased in future studies. Third, only male mice were used; including female mice will be important to assess potential sex-dependent difference. Finally, all experiments were performed at room temperature, whereas repeating them at physiological temperature (32–37 °C) would provide a more accurate reflection of *in vivo* conditions.

## Data Availability

The raw data supporting the conclusions of this article will be made available by the authors, without undue reservation.
