# Supplementary material for: GLP-1 selectively enhances tonic GABAA receptor-mediated currents in mouse dentate gyrus granule cells of the ventral hippocampus
Source: Front Cell Neurosci. 2025 Oct 1;19:1638550. doi: 10.3389/fncel.2025.1638550 (PMC12521440; doi:10.3389/fncel.2025.1638550)
Supplement: Supplementary file 1 [file Data_Sheet_1.docx]

**Supplementary information**

**
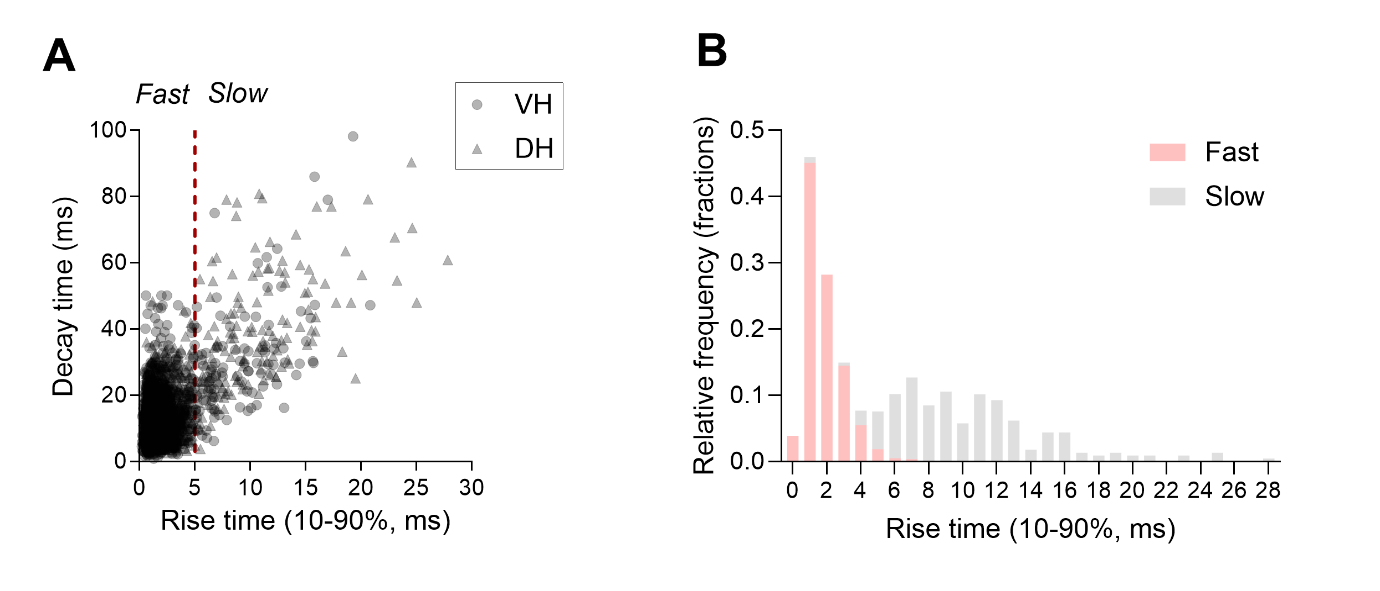
**

**Supplementary Figure 1.** **Classification of fast and slow sIPSCs recorded in DG granule cells of two-month-old mice**.

**A.** Scatter plot of 10-90% rise time versus 63% decay time for all detected sIPSCs during ACSF perfusion (n=2283). sIPSCs were classified as fast or slow events based on a 5 ms rise-time cutoff. **B.** Histogram illustrating the distribution of sIPSC rise time (10-90%) for all fast (n=2056) and slow (n=227) events during ACSF perfusion.
